# 948. Impact of a Multi-pronged Approach to Antimicrobial Stewardship on the Vascular Ward at a Tertiary Hospital

**DOI:** 10.1093/ofid/ofac492.791

**Published:** 2022-12-15

**Authors:** Maggie O Wong, Wendy L Bowles, Kevin Lee, Kevin Afra

**Affiliations:** Fraser Health, Burnaby, British Columbia, Canada; Fraser Health, Burnaby, British Columbia, Canada; Fraser Health, Burnaby, British Columbia, Canada; Fraser Health, Burnaby, British Columbia, Canada

## Abstract

**Background:**

Up to 50% of inpatient antibiotic use may be inappropriate, and guideline concordance for antibiotic prescribing in surgical wards tends to be lower than medical wards. As such, our study describes a multi-pronged approach to antimicrobial stewardship (AMS) targeting a vascular ward and its impact on improving antibiotic use.

**Methods:**

This is a quality improvement study at a tertiary hospital in British Columbia, Canada. All patients admitted to the vascular surgery ward for any vascular related issues and who received piperacillin-tazobactam or a carbapenem were included. Patients were excluded if admitted to the ward for < 48 hours. The primary outcome is appropriateness of carbapenem and piperacillin-tazobactam use in concordance with local policies. Secondary outcome is consumption of these agents.

The pre-intervention period was March 2020 to June 2020. The AMS team prospectively reviewed included patients to establish the baseline level of appropriate antibiotic use. The intervention period was from July 2020 to February 2021.

Aside from presentations to surgeons, the main intervention was regular audit and feedback to a nurse practitioner who rounds with the surgeons. Mandatory ID consultation for carbapenems was the last resort if AMS recommendations were not accepted.

**Results:**

During the pre-intervention period, 19 out of 25 prescriptions (76%), either carbapenem or piperacillin-tazobactam, had appropriate indications. In the intervention period, 121 prescriptions were reviewed and the appropriateness level increased to 87% (Figure 1). Eleven mandatory ID consultations were generated. The mean days of therapy (DOT) per 1000 patient-days for carbapenem decreased from 94.6 to 56.6 for pre- and intervention periods, respectively. For piperacillin-tazobactam, the mean DOT per 1000 patient-days decreased from 209.9 to 138.5 for pre- and intervention periods. The reduced usage of piperacillin-tazobactam remained sustainable post-intervention (Figure 2).

**Figure 1:**
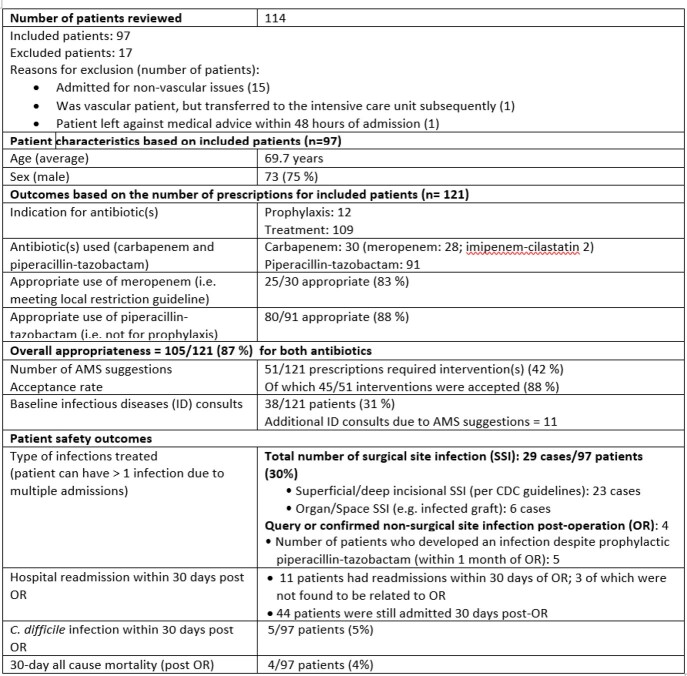
Summary of patient characteristics and outcomes during intervention period (July 2020 to February 2021)

**Figure 2:**
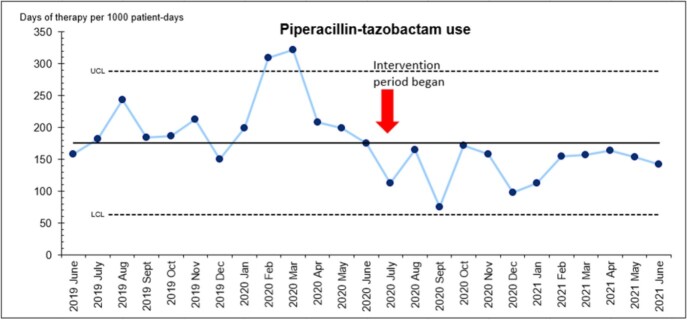
Usage of piperacillin-tazobactam from June 2019 to June 2021

**Conclusion:**

A multi-pronged approach, consisting of education, prospective audit and feedback to the surgical team, and mandatory ID consult in selected cases, is effective in decreasing inappropriate broad-spectrum antibiotic use on the vascular ward at a tertiary centre.

**Disclosures:**

**All Authors**: No reported disclosures.

